# Local regulatory mechanism to coordinate colorectal motility in rats

**DOI:** 10.14814/phy2.13710

**Published:** 2018-05-20

**Authors:** Rika Sawada, Hiroyuki Nakamori, Kiyotada Naitou, Kazuhiro Horii, Yuuki Horii, Hiroki Shimaoka, Takahiko Shiina, Yasutake Shimizu

**Affiliations:** ^1^ Laboratory of Veterinary Physiology Faculty of Applied Biological Sciences Gifu University Gifu Japan; ^2^ Department of Basic Veterinary Science Laboratory of Physiology The United Graduate School of Veterinary Sciences Gifu University Gifu Japan; ^3^ Center for Highly Advanced Integration of Nano and Life Sciences (G‐CHAIN) Gifu University Gifu Japan

**Keywords:** Colitis, colorectal motility, defecation, enteric nervous system, lumbosacral defecation center

## Abstract

The presence of a fecal pellet in the colorectum causes ascending contraction and descending relaxation, propelling the pellet aborally. However, random occurrence of the reflexes at multiple sites would disturb sequential excretion of the pellets, resulting in inefficient defecation. Hence, we postulated that a regulatory mechanism to coordinate peristaltic motility initiated at adjacent portions of the colorectum may exist. Colorectal motility was recorded with balloons located at 2 cm, 5 cm and 7 cm from the anus in vivo in anesthetized rats. The presence of a balloon in the colorectum inhibited motility of the oral side and enhanced motility of the anal side. Both the ascending inhibitory and descending facilitatory actions were unaffected by cutting the pelvic nerves, suggesting little contribution of the lumbosacral defecation center. In contrast, disrupting the continuity of the enteric nervous system abolished the local reflex mechanism. The ascending inhibitory pathway operated in a condition in which facilitatory input from the lumbosacral defecation center was fully activated by intrathecal injection of ghrelin. We also found that functional impairment of the local reflex pathways was evident in rats that recovered from 2,4,6‐trinitrobenzensulfonic acid‐induced colitis. These results demonstrate that an intrinsic regulatory mechanism to coordinate peristaltic motility initiated at adjacent portions exists in the rat colorectum. The regulation may be beneficial to propel multiple pellets efficiently. In addition, impairment of the local regulatory mechanism might be involved in postinflammatory dysmotility in the colorectum.

## Introduction

Colorectal motility is controlled by the enteric nervous system (ENS) and the central nervous system (CNS) (Furness 2006; Olsson and Holmgren 2011; Browning and Travagli 2014). The ENS is composed of primary afferent neurons, interneurons, and motor neurons, and it is able to function independently from the CNS (Furness 2006; Olsson and Holmgren 2011). Efferent neurons derived from the CNS exert their effects on motility by modulating the activity of the ENS (Olsson and Holmgren 2011; Browning and Travagli 2014). These nervous systems cause coordinated peristalsis and intraluminal feces are propelled forward to the anus. The sensory neurons of the ENS recognize the colorectal distention evoked by feces causing ascending contraction and descending relaxation via interneurons and motor neurons (Furness 2006; Olsson and Holmgren 2011). Since the polarized neural pathway plays pivotal roles in peristaltic motility, the underlying mechanism has been of particular interest (Spencer et al. 2016).

When several fecal pellets reside in the colorectum, peristaltic reflexes might occur in response to each fecal pellet simultaneously in different parts of the colorectum. Random occurrence of peristaltic reflexes at multiple sites would disturb sequential excretion of the pellets, resulting in inefficient defecation. To avoid unfavorable outcomes, a number of reflex pathways within the colorectum (e.g., colocolonic reflex and rectocolonic reflex) are known to be involved in modulation of motility (Kreulen and Szurszewski 1979; Hughes et al. 1999; Bampton et al. 2002; Law et al. 2002; Chen et al. 2010). For instance, rectal distension reduces proximal colonic motor activity (Bampton et al. 2002; Law et al. 2002). Such a reflex regulation triggered by stimulation of a distant portion of the gut is mediated by extrinsic autonomic neural pathways (Kreulen and Szurszewski 1979; Hughes et al. 1999; Chen et al. 2010).

We postulated that, in addition to the coordinating regulation between distant portions, a regulatory mechanism to coordinate peristaltic motility initiated at adjacent portions of the colorectum may exist. To address the putative mechanism, we used an in vivo experimental system for recording colorectal motility in anesthetized rats. This is because the in vivo system is suitable for assessing not only the role of the ENS but also that of the CNS. Our results suggest that the presence of a fecal pellet at a particular colorectal portion suppresses propulsion of pellets located at the oral side and enhances it at the anal side through mediation of the ENS. We also provide evidence that dysfunction of the coordinating mechanism is involved in postinflammatory dysmotility.

## Materials and Methods

### Animals

Male Sprague‐Dawley rats (Japan SLC, Inc., Shizuoka, Japan) weighing 300–450 g were used. The rats were maintained in plastic cages at 22°C with a 12:12 light : dark cycle (lights on at 06:00–18:00 h) and they were supplied with both laboratory chow (MF, Oriental Yeast Co., Ltd., Tokyo, Japan) and water ad libitum prior to experiments. The experimental procedures were approved by the Animal Care and Use Committee of Gifu University (permission number: 17011) and were performed according to the guidelines for the care and use of laboratory animals.

### Recordings of colorectal motility in vivo

Sedation was achieved with ketamine hydrochloride (50 mg kg^−1^, i.m.), followed by anesthesia with *α*‐chloralose (60 mg kg^−1^, into the tail vein). The femoral artery was cannulated and anesthesia was maintained by intra‐arterial infusion of *α*‐chloralose (10–20 mg kg^−1^ h^−1^) combined with ketamine hydrochloride (3–5 mg kg^−1^ h^−1^) in 0.9% saline. The arterial cannula was connected to a pressure transducer for recording arterial blood pressure. Body temperature was maintained at 36–37°C by a heating lamp throughout the experiments. At the end of the experiments, the rats were immediately killed by intravenous administration of a lethal dose of sodium pentobarbitone (100 mg kg^−1^, i.p.) while they were still under anesthesia.

Colorectal motility was recorded with water‐filled balloons located at 2, 5 and 7 cm from the anus. Each balloon was attached to a flexible polyethylene tube connected to a pressure transducer. All fecal pellets in the colorectum were flushed by warm saline. The most proximal (7 cm) and middle (5 cm) balloons were inserted through the cecum and the tube was anchored to the proximal colon. The most distal (2 cm) balloon was inserted through the anus and anchored to the tail. In some experiments in which the middle balloon was added later, the middle balloon was inserted through the anus. Each balloon was filled with 0.3 mL of water. The diameter of the balloon with 0.3 mL of water was about 1 cm. In this condition, the balloon did not generate pressure due to its elastic properties unless it was inserted into the colorectum. When necessary, the pelvic nerves were cut before inserting the balloons.

For application of drugs into the lumbosacral spinal cord, a 30‐gauge needle connected to a polyethylene tube was inserted between the L1 and L2 vertebrae from the dorsal surface, until tail flick appeared (L1–L2 corresponding to spinal cord level L6‐S1 in the rat). The cannula was secured in place with instant adhesive (Aron Alpha Extra; Toagosei, Co., Ltd., Tokyo, Japan) to create a tight seal at the point of cannulation. There was no cerebrospinal fluid leak.

After the surgical operation, rats were kept for about 1 h to allow basal colorectal motility and blood pressure to stabilize.

### Induction of colitis

Colitis was induced by a method described previously with slight modifications. Five‐week‐old male Sprague‐Dawley rats weighing 150–200 g were anesthetized with isoflurane. Then they were given 1 mg of 2,4,6‐trinitrobenzensulfonic acid (TNBS) dissolved in 0.6 mL of 40% ethanol (v v^−1^) by means of a silicon catheter inserted 5 cm through the anus. The animals were maintained in a head‐down position for about 1 min to prevent leakage of the intracolonic solution. The animals were maintained in their plastic cages and used at 30 days after receiving TNBS.

### Reagents

The following reagents were used: ketamine hydrochloride (Daiichi Sankyo Co., Ltd., Tokyo, Japan), *α*‐chloralose (Nakalai Tesque, Kyoto, Japan), ghrelin (Acros Organics, New Jersey, USA), and TNBS (WAKO, Osaka, Japan). Ghrelin was dissolved in distilled water. Alpha‐chlorarose was solubilized with 10% 2‐hydroxypropyl‐*β*‐cyclodextrin (Wako, Osaka, Japan) and then made up with 0.9% saline for infusion.

### Statistical analyses

Data are expressed as means ± SD. Statistical analyses of two paired groups were performed, using parametric paired, 2‐tailed Student's *t* tests. *P*‐values <0.05 were considered to be statistically significant. The area under the curve (AUC) was calculated using data of pressure changes for a period of 10 minutes.

## Results

### Effects of the middle balloon on motility of the proximal and distal parts of the colorectum

**Figure 1:**
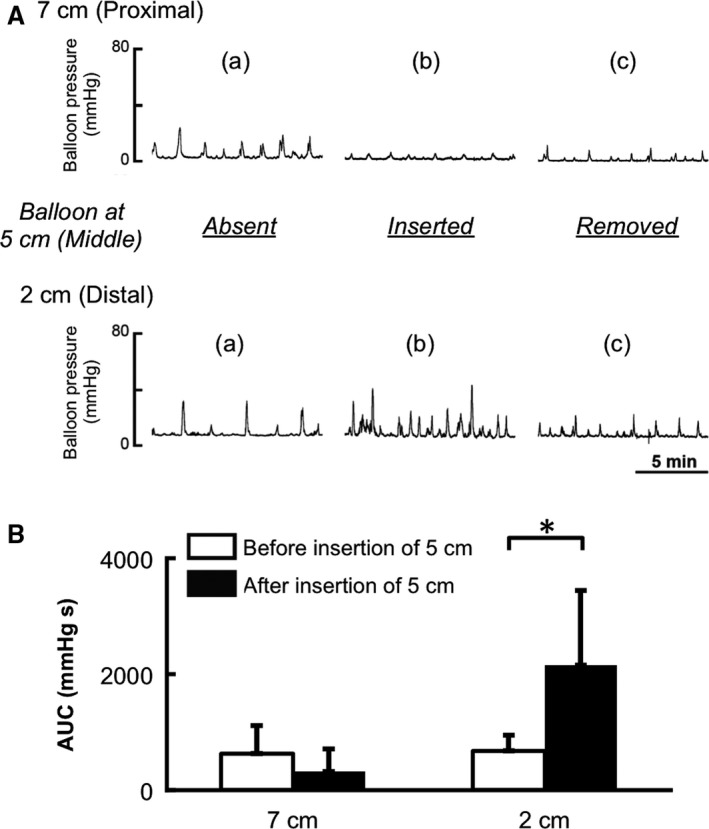
Effects of insertion of the middle balloon on motility of the proximal and distal parts of the colorectum. (A) Representative recording traces of pressure changes in balloons located at 7 cm (Proximal) and 2 cm (Distal) from the anus. Pressure changes before insertion of a balloon at 5 cm (Middle) (a), after the insertion (b), and after subsequent removal of the balloon (c) are shown. (B) Bar graph summarizes the area under the curve (AUC; mmHg s) of changes in balloon pressure before and after insertion of the middle balloon. Each value represents the mean ± SD (*n* = 5). *Significantly different at *P* < 0.05.

The balloons in the colorectum were located at 2 cm (distal), 5 cm (middle) and 7 cm (proximal) from the anus. To determine effects of pressure stimulation at a specific colorectal part on motility, we first used the middle balloon as stimuli while pressure changes were recorded from distal and proximal balloons. In the absence of the middle balloon, brief and small rises in balloon pressure occurred at the distal and proximal parts of the colorectum (Fig. 1A: a). Insertion of the middle balloon enhanced motility at the distal part (Fig. 1A, 2 cm: b). The AUC was significantly increased by insertion of the middle balloon (Fig. 1B). The enhanced motility returned to the basal level after removing the middle balloon (Fig. 1A, 2 cm: c). On the other hand, motility at the proximal part was not significantly influenced by insertion of the middle balloon (Fig. 1B).

### Effects of the distal balloon on motility of the proximal and middle parts

**Figure 2:**
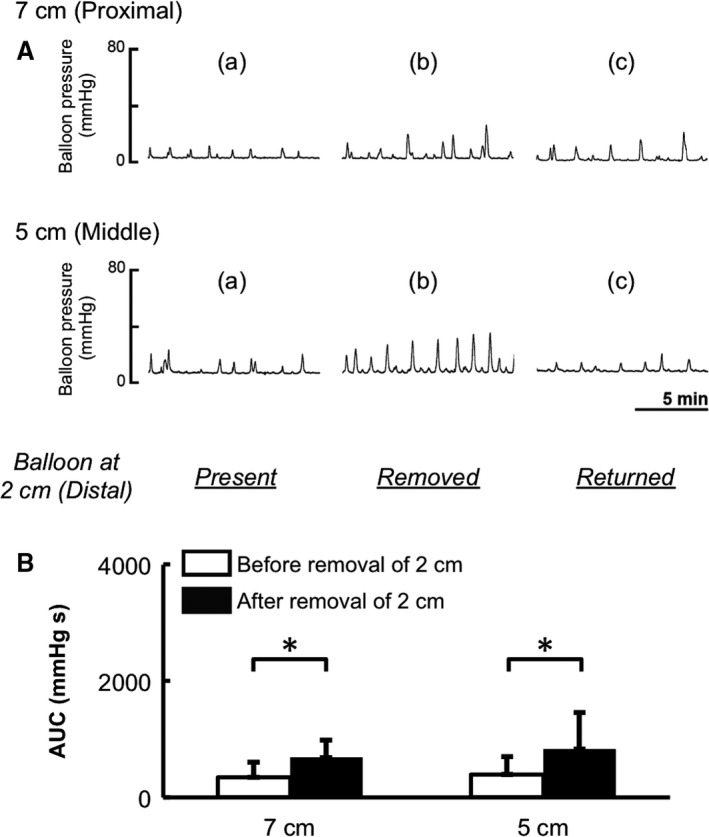
Effects of removal of the distal balloon on motility of the proximal and middle parts of the colorectum. (A) Representative recording traces of pressure changes in balloons located at 7 cm (Proximal) and 5 cm (Middle) from the anus. Pressure changes before removal of a balloon at 2 cm (Distal) (a), after the removal (b), and after subsequent reinsertion of the balloon (c) are shown. (B) Bar graph summarizes AUC (mmHg s) of changes in balloon pressure before and after removal of the distal balloon. Each value represents the mean ± SD (*n* = 5). *Significantly different at *P* < 0.05.

Although insertion of the middle balloon did not have a significant effect on motility of the proximal part, pressure changes were suppressed to some extent (see Fig. 1A, 7 cm: a vs. b). To reveal the presence of the ascending inhibitory effect from the anal side to the oral side, pressure changes in the proximal and middle balloons were recorded before and after removal of the distal balloon. When the distal balloon was present, brief and small rises in balloon pressure were observed at the proximal and middle parts of the colorectum (Fig. 2A: a). Removal of the distal balloon enhanced motility at the proximal and middle parts (Fig. 2A: b) with significant increases in the AUC (Fig. 2B). The enhanced motility returned to the basal level after reinserting the distal balloon (Fig. 2A: c).

### Role of pelvic nerves in the interactions among colorectal parts

**Figure 3:**
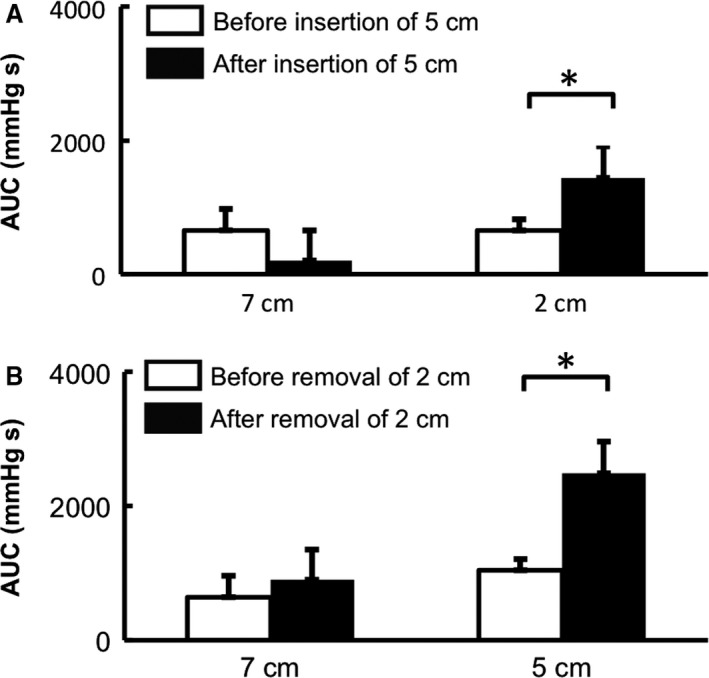
Effects of surgical transection of the pelvic nerves on the interactions among colorectal parts. Bar graph summarizes AUC (mmHg s) of changes in balloon pressure at 7 cm and 2 cm before and after insertion of the middle (5 cm) balloon (A) and changes in balloon pressure at 7 cm and 5 cm before and after removal of the distal (2 cm) balloon (B) in a rat whose pelvic nerves were transected. Each value represents the mean ± SD (A, *n* = 5; B, *n* = 6). *Significantly different at *P* < 0.05.

To examine whether the sacral parasympathetic nerves play an essential role in the interactions among colorectal parts, the pelvic nerves, which are derived from sacral parasympathetic preganglionic neurons, were bilaterally transected. Similar to observations shown in Figure 1, insertion of the middle balloon enhanced motility at the distal part compared with that before insertion of the balloon (Fig. 3A, 2 cm). The AUC for the proximal part did not significantly change compared with that before insertion of the balloon (Fig. 3A, 7 cm).

In an experiment similar to that for which results are shown in Figure 2, removal of the distal balloon significantly enhanced motility at the middle part of the colorectum compared with that before removal of the balloon (Fig. 3B, 5 cm). In contrast, motility at the proximal part was not enhanced by removal of the distal balloon (Fig. 3B, 7 cm).

### Effects of discontinuation of the ENS on the interactions among colorectal parts

**Figure 4:**
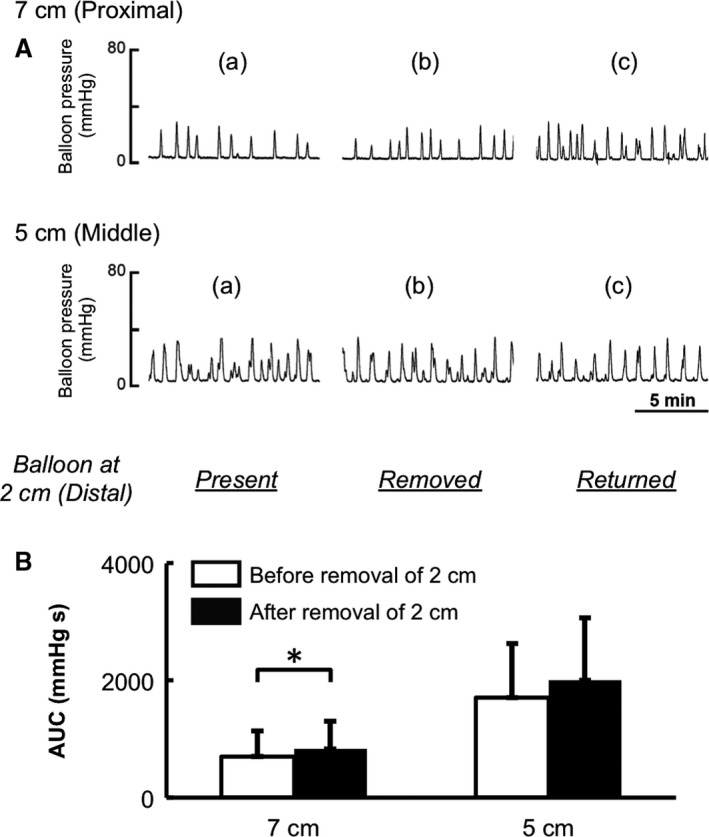
Effects of disrupting continuity of the enteric nervous system on the interactions among colorectal parts. (A) Representative recording traces of pressure changes in balloons located at 7 cm (Proximal) and 5 cm (Middle) in a rat whose colorectum was ligatured at 4 cm from the anus. Pressure changes before removal of a balloon at 2 cm (Distal) (a), after removal of the distal balloon (b), and after subsequent reinsertion of the balloon (c) are shown. (B) Bar graph summarizes AUC (mmHg s) of changes in balloon pressure before and after removal of the distal (2 cm) balloon. Each value represents the mean ± SD (*n* = 6). *Significantly different at *P* < 0.05.

**Figure 5:**
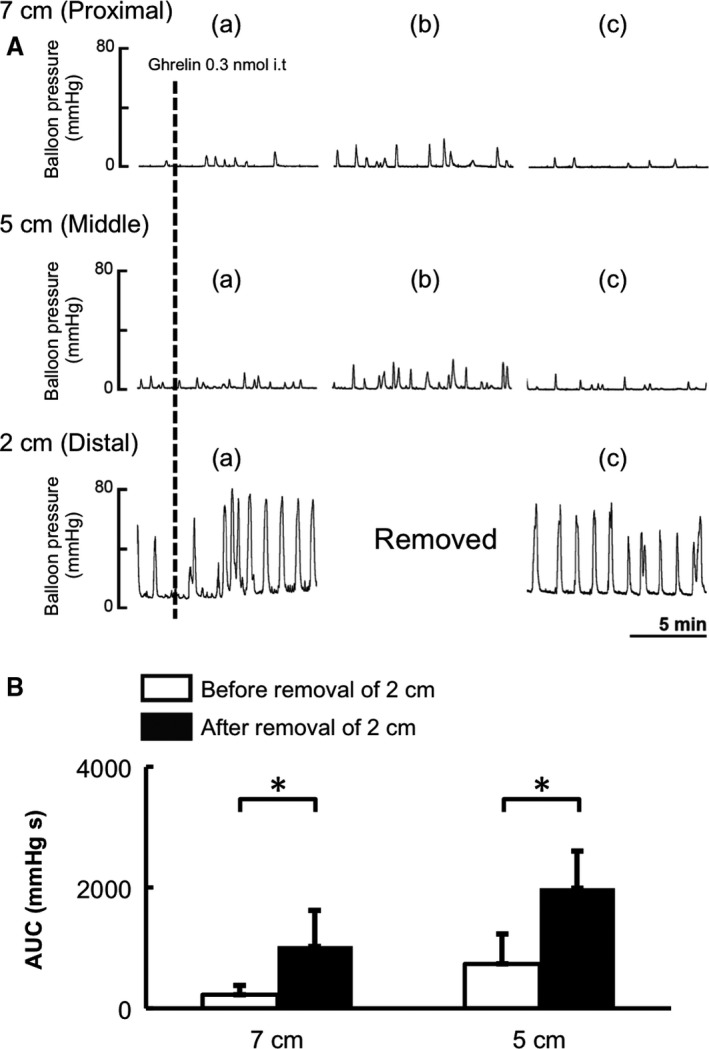
Effects of removal of the distal balloon on motility of the proximal and middle parts of the colorectum after intrathecal administration of ghrelin. (A) Representative recording traces of pressure changes in balloons located at 7 cm (Proximal), 5 cm (Middle) and 2 cm (Distal) in a rat in which ghrelin was administered intrathecally (i.t) to the lumbosacral spinal cord. Pressure changes before removal of a balloon at 2 cm (Distal) (a), after the removal (b), and after subsequent reinsertion of the balloon (c) are shown. (B) Bar graph summarizes AUC (mmHg s) of changes in balloon pressure at 7 cm and 5 cm before and after removal of the distal balloon. Each value represents the mean ± SD (*n* = 6). *Significantly different at *P* < 0.05.

We next examined roles of the ENS in the interactions among colorectal parts. To disrupt continuity of the ENS, the colorectum at 4 cm from the anus was ligatured. In rats after ligation, pressure changes at the middle part were greater in amplitude and frequency compared with those observed in intact rats (compare Figs. 2A, 5 cm: a and 4A, 5 cm: a). The AUC after the ligation was significantly higher than that without ligation (397 ± 309 mmHg s in intact vs. 1997 ± 1093 mmHg s after ligation). Motility at the proximal part was not significantly changed by the ligation (Fig. 2A, 7 cm: a vs. Fig. 4A, 7 cm: a).

In contrast to that in intact rats (see Fig. 2), motility at the middle part did not increase even after removal of the distal balloon (Fig. 4A, 5 cm: a vs. b). The AUC for the proximal part was slightly but significantly increased by removal of the distal balloon (Fig. 4B, 7 cm). After reinserting the distal balloon, motility at the proximal and middle parts was unchanged (Fig. 4A: b vs. c).

### Interaction among colorectal parts under a condition in which colorectal motility is enhanced by activation of the lumbosacral defecation center

In previous studies, we showed that activation of the lumbosacral defecation center causes strong propulsive motility of the colorectum (Shimizu et al. 2006; Naitou et al. 2015, 2016, 2018; Nakamori et al. 2018). We therefore examined whether the distal part exerts an inhibitory effect on the oral side of the colorectum even under a condition in which colorectal motility is enhanced by activation of the lumbosacral defecation center. Intrathecal injection of ghrelin (0.3 nmol) greatly enhanced motility at the distal part, whereas motility at the middle and proximal parts was unaffected (Fig. 5A: a). Fifteen minutes after injection of ghrelin, removal of the distal balloon enhanced motility at the proximal and middle parts compared with that before removal of the balloon (Fig. 5A, 7 cm and 5 cm: a vs. b). The enhanced motility returned to a level comparable to that before removal of the distal balloon after reinserting the distal balloon (Fig. 5A, 7 cm and 5 cm: c). The effect of ghrelin continued throughout the experiment because motility at the distal part was still enhanced after the balloon had been reinserted (Fig. 5A, 2 cm: c).

Since ghrelin administration to the lumbosacral spinal cord enhanced motility of the distal part maximally, it was expected that descending facilitatory action from the middle part could not be analyzed. Therefore, experiments in which the middle balloon was inserted were not carried out in this study.

### Interactions among colorectal parts after resolution of TNBS‐induced colitis

**Figure 6:**
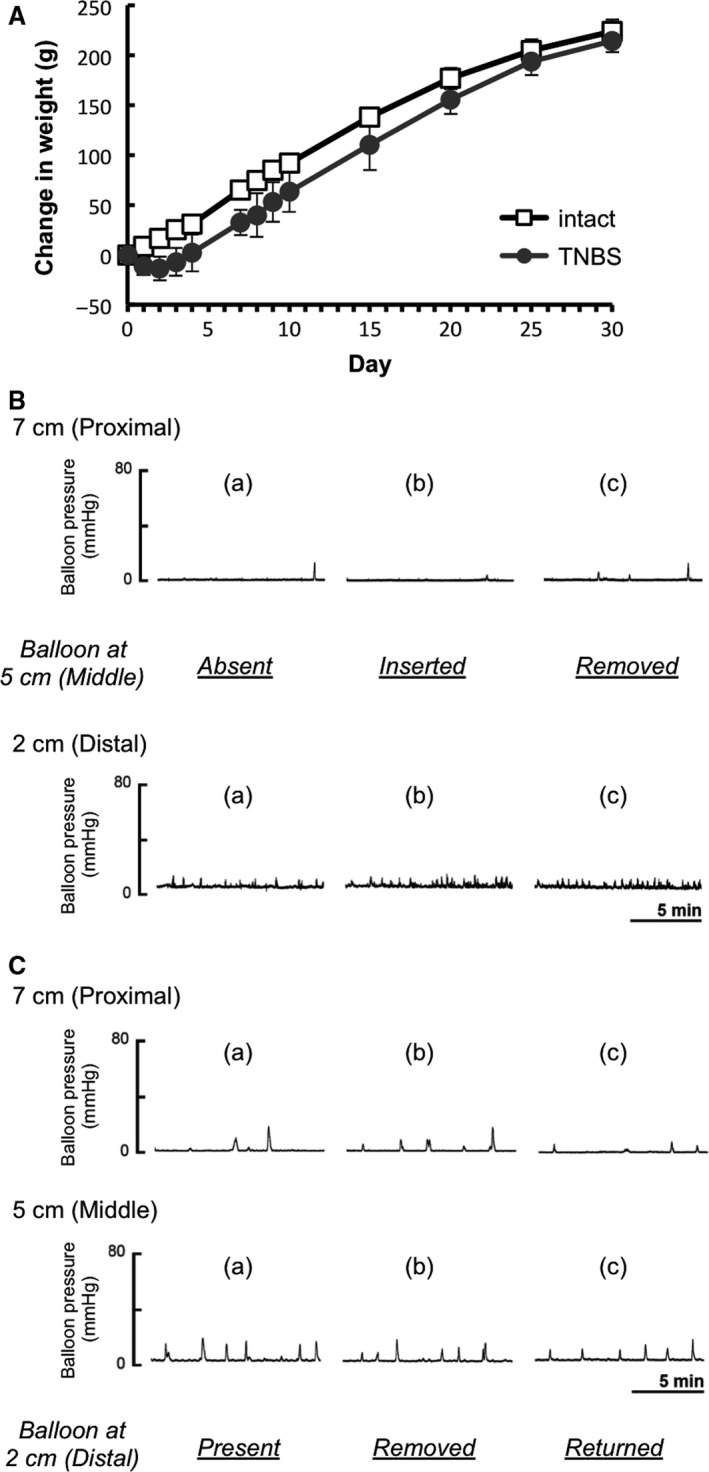
Functional impairment of the local reflex pathways after resolution of 2,4,6‐trinitrobenzensulfonic acid (TNBS)‐induced colitis. (A) Body weight changes in TNBS‐treated and intact rats (*n* = 5). At day 0, TNBS was injected into the distal colon. Each value represents the mean ± SD. (B) Representative recording traces of pressure changes in balloons located at 7 cm (Proximal) and 2 cm (Distal) in a rat that recovered from the colitis (30 days after TNBS treatment). Pressure changes before insertion of a balloon at 5 cm (Middle) (a), after the insertion (b), and after subsequent removal of the balloon (c) are shown (*n* = 5). (C) Representative recording traces of pressure changes in balloons located at 7 cm (Proximal) and 5 cm (Middle) in a rat that recovered from the colitis. Pressure changes before removal of a balloon at 2 cm (Distal) (a), after the removal (b), and after subsequent reinsertion of the balloon (c) are shown (*n* = 5).

Patients with inflammatory bowel disease (IBD) and animal models of intestinal inflammation have gastrointestinal dysmotility (Linden et al. 2003; Sands 2004; Shiina et al. 2013; Haase et al. 2016). In addition, it has been reported that persistent alterations in gastrointestinal motility continued even after resolution of intestinal inflammation (Isgar et al. 1983; Mawe 2015). We examined whether disruption of the interactions among colorectal parts is attributable to the long‐lasting alteration of motility by using a colitis model in rats. After treatment with TNBS, body weights decreased in the initial 2 days but recovered gradually as days passed (Fig. 6A). Diarrhea occurred 1–2 days after TNBS treatment and persisted for about 10–12 days. Macroscopic observation showed that treatment with TNBS induced visible inflammation in the colon at 7 days after TNBS treatment, while no lesion was observed after 30 days (data not shown).

In rats at 30 days after TNBS treatment, pressure changes recorded by the balloons set at three parts of the colorectum were small in amplitudes (Fig. 6B: a vs. Fig. 1A: a). In contrast to that in normal rats (see Fig. 1), insertion of the middle balloon failed to enhance motility at the distal part (Fig. 6B, 2 cm: a vs. b). The AUC for the distal part was not significantly different before and after insertion of the middle balloon (276 ± 151 mmHg s and 572 ± 566 mmHg s, respectively). Similarly, removal of the distal balloon did not facilitate motility at the middle and proximal parts (Fig. 6C, 5 cm and 7 cm: a vs. b). The AUC for the middle part and that for the proximal part were not significantly different before and after removal of the distal balloon (5 cm, 427 ± 222 mmHg s and 602 ± 373 mmHg s, respectively; 7 cm, 160 ± 146 mmHg s and 176 ± 226 mmHg s, respectively).

## Discussion

In this study, we demonstrated the presence of a regulatory mechanism to coordinate peristaltic motility initiated at adjacent portions of the colorectum in rats. The mechanism is dominantly attributable to the action of the ENS. Supportive evidence for this is: (1) application of distention stimulus at one part of the colorectum brought about enhanced motility at the anal side and suppressed motility at the oral side, (2) these regulatory actions were not affected by cutting the pelvic nerves but were abolished by disrupting the continuity of the ENS, and (3) the local regulatory mechanism can operate even in a condition in which facilitatory input from the spinal defecation center is fully activated by intrathecal injection of ghrelin. In addition, we revealed that the local regulatory mechanism is compromised in a postinflammatory condition. The fact that functional impairment of the local reflex pathways might be involved in dysmotility after resolution of colitis indicates their important physiological roles in control of colorectal motility.

It is well known that a fecal pellet causes ascending contraction and descending relaxation, resulting in peristaltic motility that transports the fecal pellet from the oral side to the anal side (Furness 2006; Olsson and Holmgren 2011). This study uncovered a regulatory mechanism by which motility triggered by a pellet at one part of the colorectum is suppressed by the presence of another pellet at a slightly distal part and is enhanced by a pellet at a proximal part. The neural pathway responsible for the regulatory mechanism differs from that mediating reflexes known so far as the colocolonic reflex or rectocolonic reflex (Bampton et al. 2002; Law et al. 2002). The reflexes between distant portions are mediated by the extrinsic autonomic neural pathways (Kreulen and Szurszewski 1979; Hughes et al. 1999; Chen et al. 2010). On the other hand, the regulatory mechanism operating between adjacent portions is dominantly mediated by the ENS. Suppression of motility at a proximal part can prevent further passage of fecal pellets to the colorectum where a pellet is still present. Acceleration of motility at a distal part enables emptying of intraluminal contents, allowing transport of the proximal pellets. Thus, the ENS plays an important role not only in induction of peristaltic motility but also in efficacious excretion of sequential multiple pellets. Cooperation of the local reflex pathways with long‐loop reflex pathways appears to contribute to overall coordination of colorectal motility.

Application of pressure by balloons often becomes painful stimuli. However, balloon pressure applied in this study was much lower than the noxious range (Winston et al. 2007; Liu et al. 2008; Hultin et al. 2012). Hence, the reflexes between adjacent colorectal parts seem to be induced in the physiological pressure range. Recent studies have shown that the speed of propulsion is influenced not only by the magnitude of stretch but more widely by the physical (consistency, shape and size) and chemical composition of the luminal contents (Bampton et al. 2002; Costa et al. 2015; Spencer et al. 2016). It is expected that dominant operation of the ascending inhibitory pathway over the descending facilitatory pathway results in reduction in the speed of propulsion, and vice versa. To elucidate possible involvement of the local reflex pathways in the content‐dependent changes in propulsive motility, further studies, especially those focusing on effects of physicochemical composition of the contents on the bidirectional local reflex pathways, are needed.

In the distal colon and rectum, where local interaction was examined in this study, motility is enhanced by the lumbosacral defecation center through the pelvic nerves (Hirayama et al. 2010; Naitou et al. 2015, 2016, 2018; Nakamori et al. 2018). The fact that both the ascending inhibition and descending facilitation was unaffected by severing the pelvic nerves (Fig. 3) indicates that the CNS including the lumbosacral defecation center plays little, if any, role in the interaction between adjacent parts. However, in experiments where continuity of the ENS was disrupted by ligation, removal of the balloon at the distal (2 cm) part slightly but significantly enhanced motility of the proximal (7 cm) part (Fig. 4). Interruption of the ENS network is evident as interaction between closer portions (i.e., 2 vs. 5 cm) was abolished by the ligation. It is therefore rational to consider that, in contrast to portions closely apposed from each other, interaction between portions slightly distant is mediated at least in part by the extrinsic neural pathways in a manner similar to colocolonic/rectocolonic reflex (Kreulen and Szurszewski 1979; Hughes et al. 1999; Chen et al. 2010). It is possible that sympathetic entero‐enteric regulatory reflex would be related to the reaction (Kuntz and Saccomanno 1944). Taken together, the results indicate that reflex pathways composed of the ENS and those related to the CNS complementarily function to coordinate peristaltic motility in multiple sites of the colorectum.

We previously reported that ghrelin administered into the lumbosacral defecation center causes strong propulsive contractions through mediation of the pelvic nerves (Shimizu et al. 2006; Hirayama et al. 2010). In this study, we found that distension of the distal part exerted an inhibitory effect on the adjacent oral part of the colorectum even under a condition in which colorectal motility is maximally enhanced by intrathecal administration of ghrelin to the lumbosacral spinal cord (Fig. 5). This indicates that the local reflex pathways can work independently of the stimulatory regulation of CNS. Our results also demonstrate that the prokinetic effect of ghrelin is primarily due to enhanced motility of the rectum, not the distal colon. This suggests that the direct action of ghrelin is just excretion of pellets located in the rectum. However, excretion of rectal pellets would inactivate the ascending inhibitory pathway and enhance motility in the proximal parts, resulting in transport of the next pellet to the rectum. As a result, ghrelin, in cooperation with the local reflex pathways, excretes many pellets as reported previously (Shimizu et al. 2006; Shafton et al. 2009).

Our findings may have an implication for the pathogenesis of gastrointestinal dysmotility, which is commonly observed in IBD patients during periods of clinical remission (Isgar et al. 1983; Mawe 2015). It has been reported that electrophysiological properties and neurotransmission of the ENS remain altered after resolution of intestinal inflammation (Krauter et al. 2007; Lomax et al. 2007; Shiina et al. 2013; Mawe 2015). The sustained alterations in enteric neural signaling are believed to be a cause of dysmotility (Lomax et al. 2007). In this study, we found that both the ascending inhibitory pathway and the descending facilitatory pathway do not operate even after resolution of colitis (Fig. 6). It is thus probable that the impairment of the local reflex pathways is involved in postinflammatory dysmotility in the colon.

In summary, we have shown the presence of an intrinsic regulatory mechanism to coordinate peristaltic motility initiated at adjacent portions of the colorectum in rats. The regulation may be beneficial to propel multiple pellets efficiently. In addition, this mechanism is impaired even after resolution of colitis, being involved in postinflammatory dysmotility in the colorectum.

## Conflict of Interest

The authors declare no conflict of interest associated with this manuscript.
